# Effect of *Helicobacter pylori* on enamel and dentin development – an *in vitro* study in mice

**DOI:** 10.1080/20002297.2025.2500670

**Published:** 2025-05-07

**Authors:** Carin Sahlberg, Eija Salmela, David P. Rice, Kazuhiko Nakano, Ryota Nomura, Satu Alaluusua

**Affiliations:** a Department of Oral and Maxillofacial Diseases, University of Helsinki and Helsinki University Hospital, Helsinki, Finland; b Department of Pediatric Dentistry, Graduate School of Dentistry, Osaka University, Osaka, Japan; c Department of Pediatric Dentistry, Graduate School of Biomedical and Health Sciences, Hiroshima University, Hiroshima, Japan

**Keywords:** *Helicobacter pylori*, dental enamel, dentin, mouse molar teeth, growth phases, infection, tissue culture, Turner teeth

## Abstract

**Objective:**

A heavy infection in a primary molar tooth can impair the enamel formation of the underlying permanent successor. *Helicobacter pylori* colonizes primarily the stomach, but it has also been detected in oral samples, including in the dental pulp of infected primary teeth. Here, we aim to test if *H. pylori* can disturb enamel and dentin formation.

**Methods:**

Mandibular molar explants of E18.5 mice were grown for 12 days in media containing 10% of *H. pylori* cell lysates. The presence and extent of enamel and dentin on the mesial surface of the first molar explants were evaluated from stereomicroscopic photographs and histologically.

**Results:**

The statistical analyses revealed that less enamel was formed in the test (N = 47) than in the control first molars (N = 28, *p* < 0.001). Most severe disturbances were seen in explants grown in media containing *H. pylori* cell lysates, which were made from stationary growth-phase cultures, with high optical density. Histological findings showed that dentin mineralization was also impaired.

**Conclusion:**

The results suggest that H. pylori disturbs enamel and dentin development in cultured mouse embryonic molar teeth. This provides new insight into the etiology of enamel disturbances in permanent teeth.

## Introduction

*Helicobacter pylori* is a gram-negative, microaerophilic, flagellated, and invasive motile rod, with a distinctive spiral shape. It colonizes gastric epithelial cells and is a strong risk factor for developing gastric complications like chronic gastritis, intestinal ulceration, and gastric cancer. Its pathogenesis and disease outcomes are mediated by a complex interplay between bacterial virulence factors, host, and environmental factors as presented in recent review articles [1–3].

Over 40% of the world’s population is infected with *H. pylori* [4]. Oral or faecal – oral transmission from person to person between family members is likely, and the bacterium is generally acquired during the first years of life [5,6]. Global prevalence in children ≤6-years old is reported to be 24% [7].

*H. pylori* primarily colonizes the stomach, but evidence suggests occasional or persistent colonization also in oral and dental tissues [8,9]. The organism has been detected in samples of dental pulp [10–12] and dental pulp, rather than dental plaque or saliva, has been shown to be the main reservoir for *H. pylori* in the oral cavity [12]. There is a close association between *H. pylori* infection in the oral cavity and dental caries [13]. *H. pylori* was detected in 70% of the samples obtained from deep carious lesions of a group of 3–6 years old Indian children [14] and from 30% of 4–7 years old children, respectively, from the United Arab Emirates [15]. *H. pylori* has also been isolated from infected root canals of primary teeth [16] and using a nested PCR system, Nomura and co-authors [11] further showed that *H.* *pylori* was present in over 40% of inflamed pulp specimens obtained from primary teeth of a group of Japanese children.

In humans, the interradicular region of the primary molar is of special significance due to its close anatomical relationship with the dental germ of the permanent successor [17]. Infection of a primary molar due to severe caries in early childhood may induce various disturbances in the successor ranging from demarcated opacities and hypoplasia of the enamel to total arrest of dental development [18–23]. This phenomenon was already noticed in 1912 by Turner [24], when he described a 3-year-old girl with heavily infected primary molar and subsequent hypoplasia in the premolar, so-called ‘Turner teeth’.

Several studies have assessed the microbiota of infected pulps and root canals in primary teeth. The data obtained have demonstrated the polybacterial nature of endodontic infections of primary teeth with a predominance of mandatory and facultative anaerobic bacteria [25]. However, although it is generally agreed that endodontic infection of a primary tooth may cause disturbances in the development of the dental hard tissues of the permanent successor, the effects of specific bacteria including *H. pylori* are unknown.

*H. pylori* infection-associated animal models have been widely used to reveal the mechanisms behind different pathological conditions [26]. As far as we know, effects of *H. pylori* on the development of dental hard tissues have so far not been reported. The tooth is an organ in which reciprocal epithelial – mesenchymal interactions regulate morphogenesis and cell differentiation [27,28]. The development is genetically controlled but subject to environmental factors which may irreversibly impair tooth formation. The first sign of tooth development is thickening of the ectoderm at the site of a future tooth. The subsequent development is divided into three phases, namely bud, cap and bell stages. In mice, ectodermal thickening at the site of the first molar is seen on embryonic day 11 (E11). Morphogenesis continues through the cap stage (E14) to the late bell stage (E18) when the basic cuspal morphology has been completed. During the mid to late bell stage, odontoblasts and ameloblasts differentiate at the interface of the epithelium and mesenchyme and start to secrete dentin and enamel mineralizing matrices, respectively. In mouse first molars, dissected on embryonic day 18 (E18.5) and cultured for 10–12 days, odontoblasts have laid down a thick (pre)dentin layer. Dentin mineralization and the subsequent formation of enamel by ameloblasts have started to proceed from the mesial surfaces of the mesial cusps.

The first aim of the present study was to find out if *H. pylori* could disturb the development of the dental hard tissues *in vitro*. Here, we used a mouse model in which the mandibular molar explants were dissected at E18.5 and cultured for 12 days in media containing 10% of *H. pylori* cell lysates. The lysates were made from liquid cultures representing different growth phases of *H. pylori*. The second aim was to see if the disturbances in the development of the dental hard tissues were connected to the growth phases of *H. pylori*.

## Materials and methods

### Ethics statement

The use of animals was in accordance with the European Convention for the Protection of Vertebrate Animals used for Experimental and Other Scientific Purposes that has been approved by the Institutional Animal Care and Use Committee (IACUC) of the Faculty of Science of the University of Helsinki. The use of animals was approved by the Institutional Animal Care and Use Committee of the University of Helsinki. Animals were provided by the Laboratory Animal Centre at Biomedicum Helsinki. The study conforms to the ARRIVE Guidelines 2.0.

### Animals and tooth culture

Pregnant mice (CD1) were anesthetized with CO_2_ and sacrificed by cervical dislocation on E18.5. The day of the vaginal plug was set as E0. Mandibular molar tooth germs from E18.5 embryos were dissected under a stereomicroscope. Tooth explants were transferred to a Trowell-type organ culture with metal grids and Nucleopore filters on small Petri dishes [29]. Each culture contained two test groups and one control group. The number of explants in the final study in each test and control group varied from 5 to 12. In total, there were 75 explants. The total number of pregnant mice was 9 in the preliminary experiments and 5 in the final study.

The tooth germs were dissected in PBS and cultured in Dubecco’s modified Eagle’s medium (D-MEM; Gibco BRL, Paisley, Scotland) supplemented with 10% of fetal calf serum (FCS; Gibco BRL), 100 μg/mL ascorbic acid (Sigma, St. Louis, MO, USA) and Penicillin-Streptomycin (PS, 20 IU/mL). This medium, D-MEM/FCS, is called here the basal medium. The level of the medium contacted the plane of the grid but did not cover it.

The explants were cultured for 12 days at 37°C in 5% CO_2_ in humidified air. The medium was changed every 2–3 days. At each change, the growth of the test and control explants was monitored under a stereomicroscope.

### *H. pylori* strain and its culture conditions

*H. pylori* strain 26695 (ATCC 700392) was purchased from Summit Pharmaceuticals International Corporation (Tokyo, Japan). The strain was grown at 37°C on blood agar plates containing 5.5% sheep blood (Oxoid Limited, Hampshire, United Kingdom) under microaerophilic conditions (CampyGen™ system; Oxoid). Liquid cultures were grown in D-MEM/FCS with gentle agitation at 120 rpm in 5% CO_2_ at 37°C. For storage, the bacteria were frozen in −80°C.

Colonial morphology on blood agar, Gram staining, light microscopy and phase contrast microscopy × 400 were used to study the characteristics of *H. pylori*.

### Construction of growth curves of *H. pylori*

**Figure 1.**
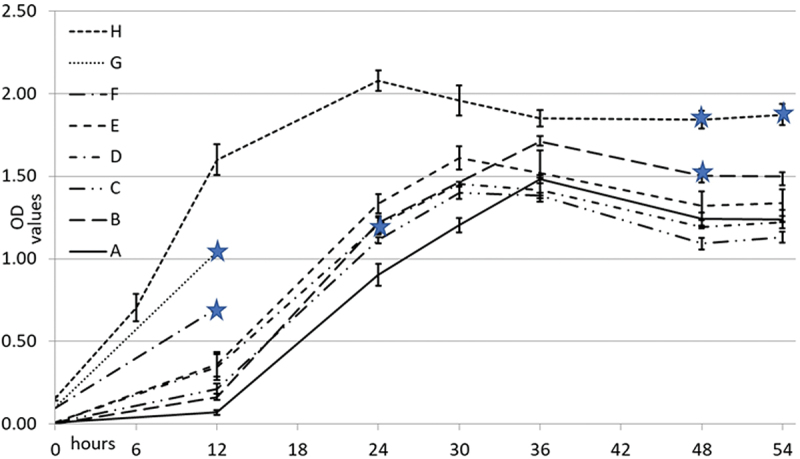
Eight growth curves of *Helicobacter pylori* 26695 and the time-points for processing the cell lysates (stars). Colonies of *H. pylori* were collected from blood agar and cultured in 10 ml of D-MEM supplemented with 10% of FCS in 5% CO_2_ at 37°C. In the curves A-G the starting colony number ranged from 1 to 20. In curve H, numerous colonies were collected resulting in OD_600_ value of 0.15 at the starting point. Each curve, except F and G, represents three independent cultures with standard deviations. Logarithmic growth phase changed to stationary growth phase at around 24 hours from the start when the number of bacteria in the inoculum was high (H) and at around 30–36 hours when lower numbers of colonies were inoculated in the culture media at the starting point.

From frozen storage, bacteria were inoculated on blood agar plates and grown for 2–4 days. Varying number of bacteria were then collected from the blood agar into tubes containing 10 ml of the basal medium. The number of colonies collected ranged from one to numerous colonies, which resulted in the highest initial optical density (OD_600_) value of 0.15. The post-inoculation growth was followed by recordings of the OD_600_ values at the time points of (6), 12, 24, 30, 36, 48 and 54 hr. Six growth curves, each based on triplicate cultures and two curves each based on one 12 hr culture were constructed (Figure 1).

### Processing of the *H. pylori* liquid cultures by lysing the cells

As for the construction of the growth curves, for the final study varying numbers of *H. pylori* colonies at the starting point were inoculated in 10 ml of the basal medium. The liquid cultures representing the logarithmic growth phase were further processed at the time points of 12 hr (OD_600_ 0.71 and 1.04) and 24 hr (OD_600_ 1.25) and those representing the stationary phase at 48 hr (OD_600_ 1.50 and 1.90) and 54 hr (OD_600_ 1.93). During processing, bacterial cells were lysed by sonication on ice, centrifuged at 4000 rpm for 10 min and passed through a 0.22 μm filter under aseptic conditions to remove any remaining bacteria. The cell lysates, stored at −20°C until use, included soluble cell-associated proteins (cytoplasmic and periplasmic proteins) in addition to those secreted outside the bacteria.

### Tooth explant cultures in the preliminary experiments and in the final study

**Figure 2.**
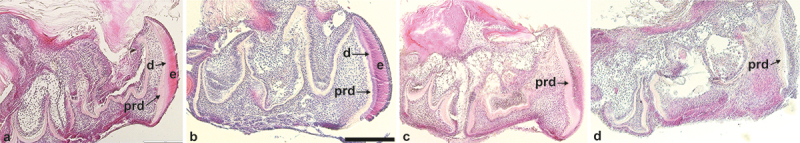
Effects of *Helicobacter pylori* cell lysate on hard tissue formation of E18.5 mandibular first molar explants in a preliminary experiment after 12 days of culture. Histological appearances of HE-stained demineralized paraffin sections. (a) Control. Explant cultured in the basal medium D-MEM/FCS. (b) Explant cultured in the basal medium containing 5% of *H. pylori* cell lysate from the logarithmic growth phase culture (OD_600_ 0.65). The cell lysate was added to the basal medium on the third day of culturing. Thick enamel layer (e) is seen on the mesial surface. Also the predentin (prd) and mineralized dentin layers (d) are present. The appearance is comparable to that of the control (a). (c) Explant cultured in the basal medium containing 10% of *H. pylori* cell lysate from the stationary growth phase culture (OD_600_ 1.90). The lysate was added to the basal medium from the beginning of the culturing. Only predentin is present. (d) Explant cultured in the basal medium containing 40% of *H. pylori* cell lysate from the stationary growth phase culture (OD_600_ 1.90). Length from cervical loop to cusp tip is reduced. Bar: 200 µm.

As mentioned, this may be the first study on the effects of *H. pylori* on tooth development. Therefore, we did several preliminary experiments before the final study. We first tested the possibility to culture the explants with living *H. pylori*. However, the growth of the bacteria was rapid and difficult to control. Therefore, *H. pylori* cell lysates were used in the preliminary experiments and in the final study. Cell lysates from the logarithmic (OD_600_ 0.65) and stationary growth-phase cultures (OD_600_ 1.90) were used in preliminary tests. To determine the percentage of *H. pylori* cell lysate in the basal medium needed to cause disturbances in the development of dental hard tissues, the percentages of 1%, 2%, 5%, 10% and 40% were tested. Also, the focus in the preliminary studies was on the time-point of the start of exposure of the explants. The tested exposure starting points were E18.5 and E18.5 + 3 days. Based on the results of the preliminary experiments (Figure 2), basal medium containing 10% of *H. pylori* cell lysate and the starting point of exposure at E18.5 were chosen for the final study, in which the number of exposed explants was 47. The control explants (N = 28) were grown in the basal medium.

At the end of the tooth culture period, 40 μl of the medium from each Petri dish were inoculated on blood agar and cultured at 37°C under microaerophilic conditions for 3 days in order to exclude possible bacterial contamination.

### Examination of the dental hard tissues

**Figure 3.**
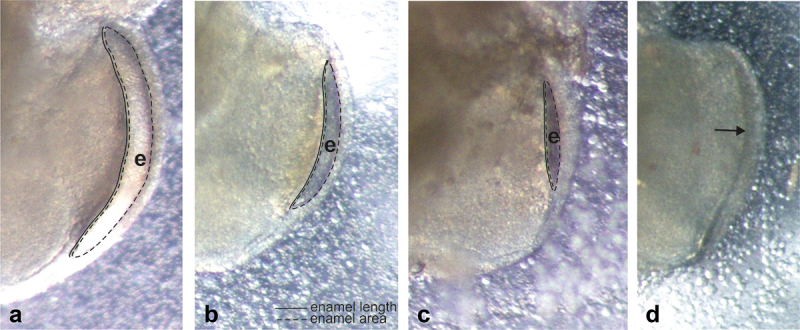
Examples of enamel presence and extent in stereomicroscopic pictures taken from the mesial surface of E18.5 mouse first molars after 12 days of culturing. (a) Score 3. The enamel layer (e) is thick and it extends from the cusp to the cervical area. In the pixel measurements the length of enamel is 495 and the enamel area 25,015. (b) Score 2. The enamel and dentin layers are thinner than in score 3. Comparable pixel values of the enamel are 310 and 7335. (c) Score 1. Enamel layer is very thin and covers only a small area. The pixel values are 222 and 4414, respectively. (d). Only predentin is present (arrowhead) and the pixel value for enamel is 0. The magnification of all the pictures is the same.

The growth and development of the dental hard tissues of the mandibular molar tooth explants that were dissected from E18.5 embryos were monitored under a stereomicroscope always when the medium was changed. After 12 days of culture, the explants were fixed with 4% paraformaldehyde, demineralized with EDTA (0.33 M/L), embedded in paraffin, serially sectioned, and stained with hematoxylin-eosin. Differences between the test explants and the controls were evaluated from the stereomicroscopic photographs taken after the culture, before fixation and the estimation was confirmed by histological analysis of representative explants. The presence and extent of enamel on the mesial surface of the first molar was evaluated as described by Salmela et al. [30]. Briefly, score 3 was given to teeth with a thick and even enamel layer extending well over halfway from the height of the mesial cusp (Figure 3(a)). Score 2 was given to teeth showing clearly thinner enamel or less extended enamel (Figure 3(b)). Score 1 was given if there was only a spot of enamel or mineralized dentin mesially (Figure 3(c)) and score 0 if there was no enamel or mineralized dentin at all (Figure 3(d)). The scores for each explant were given independently by three examiners (ES, CS, SA) who gave a score from 3 to 0 to every explant. The mean value of the scores given by the examiners was used in statistical analyses. The agreement of the scores between the examiners was determined by Kendall’s tau coefficient. In addition, enamel length and enamel area were measured from stereomicroscopic photographs using cellSens Standard 1.11 software (Olympic Corporation). The differences in scores and pixel values between the test and control explants as well as between explants grown with *H. pylori* cell lysates from the logarithmic and stationary growth-phase cultures were tested by Mann-Whitney U-test using SPSS (version 29.0.2.0 (20)) software. The probability value of <0.05 was considered significant.

## Results

### Formation of enamel and dentin in preliminary experiments

In the preliminary experiments, it became clear that the basal medium containing high concentration of *H. pylori* cell lysate (10% or 40%), which was collected at stationary growth phase from a culture with high OD_600_ value (1.90) (see Figure 1), totally inhibited enamel and mineralized dentin formation in the first molar explants cultured for 12 days (Figure 2(c,d)). In turn, enamel and dentin development of explants grown in the basal medium containing 5% of *H. pylori* cell lysate from the logarithmic growth-phase culture (OD_600_ 0.65) and added to the basal medium on E18.5 + 3 onwards, did not differ from the controls (Figure 2(a,b)).

### Development of enamel and dentin of the controls

**Figure 4.**
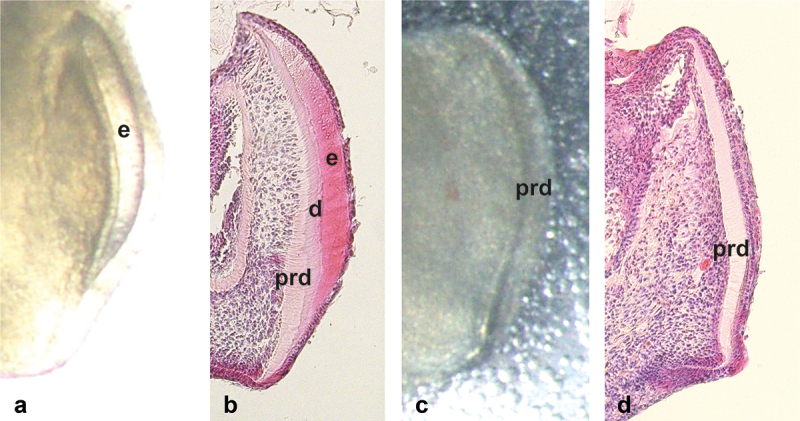
Stereomicroscopic (a,c) and histological (HE-stained demineralized paraffin sections) (b,d) appearances of the mesial surface of mouse E18.5 mandibular first molar explants after 12 days of culture. Comparison between the controls (a,b) and the explants cultured in basal medium containing 10% of *Helicobacter pylori* cell lysate from the stationary growth phase culture (OD_600_ 1.90) (c,d). In the control molar (a,b), the enamel layer (e) in the mesial surface is thick and it extends from the cusp tip to the cervical area. As seen in b, predentin (prd) and mineralized dentin (d) are distinguishable throughout the mesial surface. Due to the higher affinity of hematoxylin to the organic matrix of mineralized dentin than to predentin, mineralized dentin stains darker. The thickness of enamel is equal to or greater than the combined thicknesses of predentin and mineralized dentin. In the test molar (c,d), only predentin is clearly visible. Enamel or mineralized dentin are not present.

**Figure 5.**
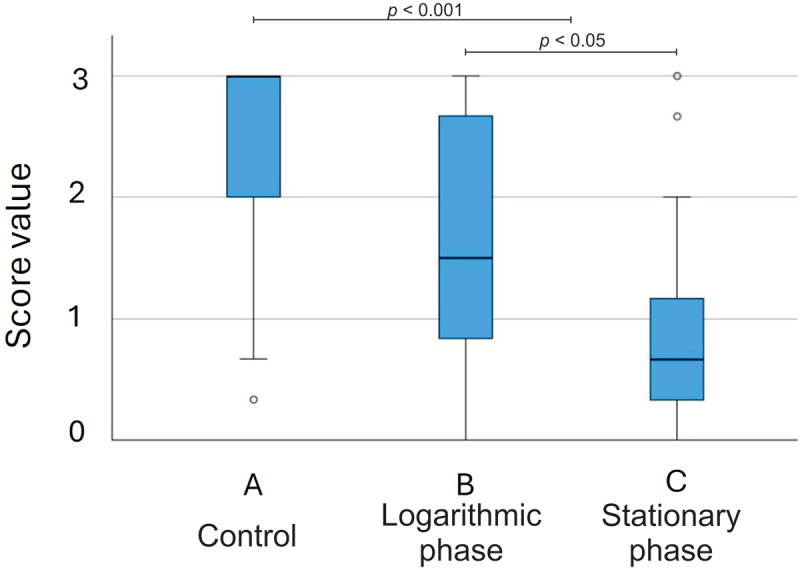
Distribution of scores indicating the presence and extent of enamel on the mesial surface of the first molar of the test and control explants. The boxplot compares the medians and the middle 50% of means of the scores of the controls (A), explants grown in media containing 10% of *H. pylori* cell lysates from the logarithmic growth phase cultures (B) and those grown in media containing 10% of *H. pylori* cell lysates from the stationary growth phase cultures (C).

After 12 days of culturing, ameloblasts and odontoblasts in the control first molars had formed a thick layer of enamel and dentin, respectively, on the mesial side of most tooth explants as seen in the histological section in Figure 2(a) and in the stereomicroscopic image in Figure 4a. Mineralized dentin and predentin were clearly distinguishable from each other as seen in the histological section in Figure 4(b). In the final study, enamel was clearly present (scores 3 and 2, Figure 3(a,b), respectively) in 23/28 control explants. The median of the mean scores given by the three examiners was 3 (Figure 5). The mean length of the mesial enamel was 302 pixels (SD 119), and the mean enamel area was 7825 (SD 4030), respectively. Deposition of the enamel matrix in the second molar was visible in one explant.

### Effect of *H. pylori* cell lysates on enamel and dentin formation

The presence of *H. pylori* cell lysate in the basal mediumaffected dentin development and slowed down or inhibited enamel formation (Figure 4). In the statistical analysis, the mean scores of the test explants and the controls differed significantly (*p <* 0.001, Figure 5). In line, enamel length and area were significantly greater in control than in the test explants (*p <* 0.001, and *p <* 0.02) where the mean length of the mesial enamel was 183 pixels (SD 169) and the enamel area 5596 (SD 6391), respectively. Explants grown in media containing *H. pylori* cell lysates from the stationary growth-phase cultures had lower mean scores (*p <* 0.05, Figure 5) and shorter enamel length (*p <* 0.05) than those from the logarithmic growth-phase cultures. The difference in the enamel area was not statistically significant (*p* = 0.06).

In explants grown in media containing *H. pylori* cell lysates originating from the late time points of 48 hr (N = 18) and 54 hr (N = 5) enamel or mineralized dentin were not present in the majority of explants (Figure 5). The median of the mean scores was 0.7. In 17/23 explants, the scores ranged from 0 to 1. The median of the mean scores was 1 (mean 1.1) among the explants grown with *H. pylori* cell lysates originating from the time point of 48 hr, and 0 (mean 0.1) among those from the time point of 54 hr. In histological sections only predentin was visible (Figure 4(d)).

Kendall’s tau coefficient test showed highly significant correlations in scores given by the examiners (0,650–0,763, *p <* 0.001) to both the test and control explants.

## Discussion

In the present study, we used two methods to assess the enamel presence and extent in tooth explants grown with *H. pylori* cell lysates. Both methods, the scoring method and the cellSens Standard software method, showed that the presence of *H. pylori* cell lysate in the basal medium in E18.5 mouse first molar explant cultures disturbed enamel and dentin formation. The effect was seen especially in explants that were grown in media containing cell lysate, which originated from the stationary-phase culture and had high OD_600_ values. In histological sections of the tooth explants, it could be seen that in these cases mineralized dentin was not present. This suggests that the *H. pylori* cell lysate containing cytoplasmic and periplasmic proteins from *H. pylori* cells as well as those proteins excreted by the bacteria affected the ability of odontoblasts to mineralize dentin, which resulted in lack of enamel formation. It is known that mineralized dentin is a prerequisite for enamel formation [27].

*H. pylori* releases a large variety of toxins, as reviewed by Sharndama and Mba [3]. Snider and coworkers [31] analyzed multiple phases of *H. pylori* growth and detected that marked differences existed in the composition of the *H. pylori* exoproteome at different time points. Some of the 74 selectively released proteins in their study were enriched in the supernatant in nearly all phases of bacterial growth, while others were enriched mainly in early or late growth phases. According to their findings, one of the virulence factors exhibiting a striking growth phase-dependent variation in proportional abundance in the culture supernatant was the vacuolating cytotoxin antigen (VacA). It was low at early time points but increased markedly at 36 hr and 48 hr time points. We found that especially the stationary phase *H. pylori* cell lysates strongly disturbed the enamel and dentin development of the mouse first molar. It is not known which toxins affect the development of the dental hard tissues and if, for example, VacA is among them.

Since it has been shown that *H. pylori* is often present in inflamed dental pulp [11], it can be speculated that *H. pylori* is among the early invaders towards the interradicular area. *H. pylori* possesses many virulence characteristics among which are its motility and its ability to adhere to host cells [3]. As a penetration route, it can use the root canals or the accessory canals directly from the infected pulp to the furcation area of a primary molar [32–34].

The prevalence of the accessory canals in primary molars varies according to different studies from around 50% to 80% [35–37]. Kumar [34], who also measured the dimensions of the canals, reported a prevalence of 57%. The diameter of the canals in his study varied from 10 to 180 µm. In comparison, the size of *H. pylori* is approximately 3.5 µm in length and 0.5 µm in width. However, in addition to the possible role of accessory canals, other factors, such as changes in the permeability of the pulpal floor due to caries may give the route for bacteria and their metabolites [38].

Mouse molar tooth has been an organ when effects of teratogens have been determined [30]. Also, the consequences on tooth development after a virus infection have been studied in mice by Jaskoll et al. [39]. Mechanisms behind the defects caused by the teratogens and the viruses are not the same, but the consequences in enamel and dentin development are quite similar. Jaskoll and coworkers showed that mouse cytomegalovirus in organ culture induced stage-dependent enamel defects and changes in the expression of genes important in normal enamel and dentin formation [40]. Interestingly, human cytomegalovirus has also been isolated from periapical lesions of deciduous teeth [41] and children with congenital cytomegalovirus infection exhibit enamel hypoplasia and hypocalcification more often than children without the virus [42]. In congenital cytomegalovirus infection, dental defects have been mainly found in primary teeth in accordance with the fact that the crowns of primary teeth develop prenatally. In turn, in ‘Turner teeth’ enamel disturbances are located mainly in premolars. According to the chronology of dental development reviewed by Smith [43] apposition of enamel and dentin starts in premolars at the age of 1.5–2.5 years and the crown formation continues up to the age of 5–6 years. Enamel hypoplasia is probable when the tooth is damaged during the first years of life, when it is at the cap, bell or secretory stage [20,21]. Later, the occurrence of demarcated opacities is possible.

## Conclusion

The present *in vitro* study in mice suggests that *H. pylori* is able to cause disturbances in dental hard tissue formation. Dentin mineralization and enamel development were impaired in E18.5 first molars cultured for 12 days with *H. pylori* cell lysate in the basal medium. Because the basic regulatory mechanisms controlling tooth development are similar in mice and humans, it can be speculated that *H. pylori*, among other organisms, may play a role in disturbing hard tissue formation seen in teeth called ‘Turner teeth’.
